# Clinical utilization of Chimeric Antigen Receptor T-cells (CAR-T) in B-cell acute lymphoblastic leukemia (ALL)–an expert opinion from the European Society for Blood and Marrow Transplantation (EBMT) and the American Society for Blood and Marrow Transplantation (ASBMT)

**DOI:** 10.1038/s41409-019-0451-2

**Published:** 2019-05-15

**Authors:** Ankit J. Kansagra, Noelle V. Frey, Merav Bar, Theodore W. Laetsch, Paul A. Carpenter, Bipin N. Savani, Helen E. Heslop, Catherine M. Bollard, Krishna V. Komanduri, Dennis A. Gastineau, Christian Chabannon, Miguel A. Perales, Michael Hudecek, Mahmoud Aljurf, Leslie Andritsos, John A. Barrett, Veronika Bachanova, Chiara Bonini, Armin Ghobadi, Saar I. Gill, Joshua A. Hill, Saad Kenderian, Partow Kebriaei, Arnon Nagler, David Maloney, Hien D. Liu, Nirali N. Shah, Mohamed A. Kharfan-Dabaja, Elizabeth J. Shpall, Ghulam J. Mufti, Laura Johnston, Elad Jacoby, Ali Bazarbachi, John F. DiPersio, Steven Z. Pavletic, David L. Porter, Stephan A. Grupp, Michel Sadelain, Mark R. Litzow, Mohamad Mohty, Shahrukh K. Hashmi

**Affiliations:** 1 Department of Hematology and Oncology, University of Texas Southwestern Medical Center, Dallas, TX USA; 2 Cell Therapy and Transplant Program, Abramson Cancer Center and the Division of Hematology and Oncology, University of Pennsylvania, Philadelphia, PA USA; 3 Fred Hutchinson Cancer Research Center, University of Washington, Seattle, WA USA; 4 Division of Pediatric Hematology-Oncology, Department of Pediatrics, University of Texas Southwestern Medical Center and Children’s Health, Dallas, TX USA; 5 Division of Hematology/Oncology, Department of Internal Medicine, Vanderbilt University Medical Center, Nashville, TN USA; 6 Center for Cell and Gene Therapy, Baylor College of Medicine, Houston Methodist Hospital and Texas Children’s Hospital, Houston, TX USA; 7 Center for Cancer and Immunology Research, Children’s National Health System, Washington, DC USA; 8 Sylvester Comprehensive Cancer Center, University of Miami Health System, Miami, FL USA; 9 Department of Laboratory Medicine and Pathology, Mayo Clinic, Phoenix, AZ USA; 10 Institut Paoli-Calmettes, Centre de Lutte Contre le Cancer; Centre d’Investigations Cliniques en Biothérapie, Université d’Aix-Marseille, Inserm CBT, 1409 Marseille, France; 11 Adult Bone Marrow Transplant Service, Department of Medicine, Memorial Sloan Kettering Cancer Center, Weill Cornell Medical College, New York, NY USA; 12 Medizinische Klinik und Poliklinik II, Universitätsklinikum Würzburg, Würzburg, Germany; 13 Oncology Centre, King Faisal Specialist Hospital and Research Centre, Riyadh, Saudi Arabia; 14 Division of Hematology, Ohio State University Wexner Medical Center, Columbus, OH USA; 15 Stem Cell Transplantation and Cellular Therapy Program, GW Cancer Center, George Washington University, Washington, DC USA; 16 Division of Hematology/Oncology/Transplantation, Department of Medicine, University of Minnesota, Minneapolis, MN USA; 17 Experimental Hematology Unit, University Vita-Salute San Raffaele and Ospedale San Raffaele, Milano, Italy; 18 Division of Oncology, Department of Medicine, Washington University School of Medicine, St Louis, MO USA; 19 Division of Hematology, Department of Medicine, Mayo Clinic, Rochester, MN USA; 20 Department of Immunology, Mayo Clinic, Rochester, MN USA; 21 Department of Stem Cell Transplantation and Cellular Therapy, The University of Texas MD Anderson Cancer Center, Houston, TX USA; 22 The Chaim Sheba Medical Center, Tel-Hashomer, Affiliated with the Sackler School of Medicine, Tel-Aviv University, Tel-Aviv, Israel; 23 H. Lee Moffitt Cancer Center and Research Institute, Tampa, FL USA; 24 Pediatric Oncology Branch, Center for Cancer Research, National Cancer Institute, National Institutes of Health, Bethesda, MD USA; 25 Division of Hematology-Oncology, Mayo Clinic, Jacksonville, FL USA; 26 Department of Haematological Medicine, King’s College, London, UK; 27 Department of Medicine, Stanford University School of Medicine, Stanford, CA USA; 28 Bone Marrow Transplantation Program, Department of Internal Medicine, American University of Beirut Medical Center, Beirut, Lebanon; 29 Experimental Transplantation and Immunology Branch, National Cancer Institute, National Institutes of Health, Bethesda, MD USA; 30 Department of Pediatrics, Children’s Hospital of Philadelphia, University of Pennsylvania, Philadelphia, PA USA; 31 Center for Cell Engineering and Immunology Program, Sloan Kettering Institute, New York, NY USA; 32 Hôpital Saint-Antoine, APHP, Sorbonne Universite, INSERM UMRs 938, Paris, France

**Keywords:** Acute lymphocytic leukaemia, Immunotherapy

## Abstract

On August 30, 2017, the U.S. Food and Drug Administration (US-FDA) approved tisagenlecleucel (KYMRIAH, *Novartis, Basel, Switzerland*), a synthetic bioimmune product of anti-CD19 chimeric antigen receptor-T cells (CAR-T), for the treatment of children and young adults with relapsed/refractory B-cell acute lymphoblastic leukemia (B-ALL). With this new era of personalized cancer immunotherapy, multiple challenges are present ranging from implementation of a CAR-T program to safe delivery of the drug, long-term toxicity monitoring and disease assessments. To address these issues, experts representing the American Society for Blood and Marrow Transplant (ASBMT), the European Group for Blood and Marrow Transplantation (EBMT), the International Society of Cell and Gene Therapy (ISCT), and the Foundation for the Accreditation of Cellular Therapy (FACT), formed a global CAR-T task force to identify and address key questions pertinent for hematologists and transplant physicians regarding the clinical use of anti CD19 CAR-T therapy in patients with B-ALL. This article presents an initial roadmap for navigating common clinical practice scenarios that will become more prevalent now that the first commercially available CAR-T product for B-ALL has been approved.

## Introduction

Chimeric Antigen Receptor (CAR) are engineered fusion proteins that combine an extracellular antigen-binding domain with one or more intracellular T-cell signaling domains. CAR-T cells (CAR-T) use gene transfer technology (such as lentiviral or retroviral vectors) to reprogram patients’ T cells to express CARs thereby re-directing their specificity, through a mechanism independent of Major Histocompatibility Complex, to target specific tumor antigens [1]. In August 2017, the United States Food and Drug Administration (US-FDA) had a landmark approval of the drug tisagenlecleucel (KYMRIAH; *Novartis, Basel, Switzerland*) for the treatment of patients up to 25 years of age with relapsed/refractory B-cell acute lymphoblastic leukemia (B-ALL). In June 2018, the Committee for Human Medicinal Products (CHMP) at the European Medicines Agency (EMA) recommended approval of tisagenlecleucel for the same indication. While the science underlying CAR-T heralds a new therapeutic era, the treatment has risk of serious adverse effects. There are many challenges facing its utilization outside clinical trials such as determining eligibility of patients to receive this treatment and the significant financial burden on the healthcare system. Moreover, many more questions have arisen regarding the future impact of CAR-T therapy on the use of allogeneic hematopoietic cell transplantation (allo-HCT), since many patients with B-ALL undergo HCT.

Commissioned by the American Society for Blood and Marrow Transplant (ASBMT) Practice Guidelines Committee and the Acute Leukemia Working Party of the European Group for Blood and Marrow Transplantation (EBMT), a global CAR-T Task Force was developed to help identify and address the key challenges in the treatment and management of patients with relapsed or refractory B-ALL destined to undergo treatment with anti CD19 CAR-T such as tisagenlecleucel. The Task Force included representatives from ASBMT, EBMT, the International Society of Cell and Gene Therapy (ISCT), and the Foundation for the Accreditation of Cellular Therapy (FACT). The Task Force committee met twice (2017 and 2018) and identified 10 key clinical practice questions and/or clinical scenarios relevant to clinical hematologists and allied health practitioners regarding the use of CAR-T in B-ALL. Practice guidelines from this initiative for each particular question forms the basis of this article and lays out a roadmap of common issues encountered with planning and delivering CAR-T therapy for B-ALL with reference to both the commercially available products and on clinical trials.

## Where and when should patients be referred for CAR-T in B-cell acute lymphoblastic leukemia?

Tisagenlecleucel (KYMRIAH^TM^) is the first CAR-T therapy approved for treatment of children and young adults with refractory or in second or later relapse of B-ALL [2]. For safe delivery of CAR-T therapy, a robust clinical infrastructure is required to handle the complex scheduling logistics, maintain the chain-of-custody and chain-of-identity of the cellular product, and facilitate communication to manage potentially severe toxicities. Several organizations are involved in standardization and regulation of this process. The FDA oversees approval and regulation of the products, manages adverse event reporting, and supervises Risk Evaluation and Mitigation Strategy (REMS) process in partnership with the manufacturers. FACT provides standards for handling, processing, and tracking of immune effector cells, and in collaboration with The Joint Accreditation Committee ISCT-Europe and EBMT (JACIE), provides oversight of the blood and marrow transplant unit around the globe. The Center for International Blood and Marrow Transplant Research (CIBMTR) and EBMT functions as a data repository and a conduit between centers and the regulatory authorities for transplant outcomes reporting. Finally, manufacturers themselves inspect apheresis and storage facilities, and authorize individual centers to provide therapeutics. To fulfill these robust requirements for a safe delivery of CAR-T, currently only selected larger institutions are selected for performing CAR-T therapy, and these are typically centers with allo-HCT experience as they are already performing similar process for HCT [3].

**Fig. 1:**
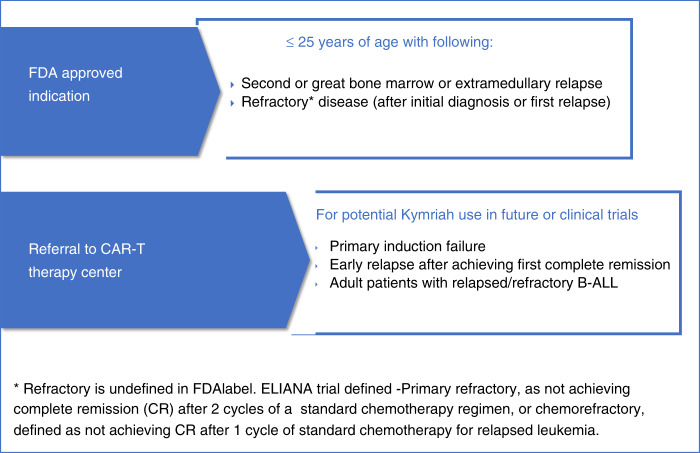
Indications for referral to center performing CAR-T therapy for evaluation of relapsed/refractory ALL

In Fig. 1, we suggest various indications for referral to centers offering CAR-T for treatment of relapsed/refractory B-ALL. In brief, tisagenlecleucel is FDA approved for patients 25 years and younger with B-ALL who have experienced a second or greater bone marrow or extra medullary relapse or have refractory disease after initial diagnosis or after treatment for first relapse. In addition, we suggest referring patients with induction failure, early relapse after achieving first complete remission, and adult patients with relapsed/refractory B-ALL to CAR-T cell therapy programs to allow discussion of the optimal timing of apheresis and potential of enrollment in CAR-T clinical trials.

**Fig. 2:**
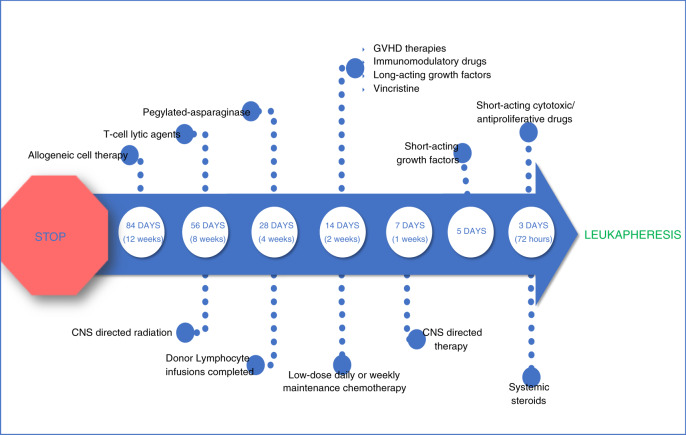
Recommended timing to stop therapies prior to leukapheresis. (GVHD graft versus host disease, CNS central nervous system)

It is important to note that most patients treated on clinical trials of anti CD19 CAR-T therapy to date have had active disease at the time of enrollment and that this therapy is effective in patients who are not in remission, which is distinct from allo-HCT for B-ALL. Thus, we recommend prompt referral to a CAR-T center as soon as a patient meets referral criteria (e.g., at the time of relapse, before starting therapy if possible,) especially as specified recovery periods from prior therapy are required before leukapheresis (Fig. 2). This is particularly important as evidence suggests that the quality of circulating T-cells decreases with increasing prior chemotherapy exposure [4]. Patients without high peripheral disease burden and sufficient circulating T-cells (and absolute lymphocyte count of >500 cells/uL or a peripheral blood CD3 count of >150 cells/uL) may be able to undergo leukapheresis for tisagenlecleucel before starting therapy for relapse. For other patients, bridging therapy should be planned in conjunction with the CAR-T therapy center to avoid therapies (in particular clofarabine) which are likely to significantly impair lymphocyte number and/or function in order to allow successful leukapheresis for CAR-T cell therapy.

## Is the sequence of CAR-T therapy with blinatumomab and/or inotuzumab important?

Blinatumomab is a bispecific CD19-directed CD3 T-cell engager indicated for the treatment of relapsed or refractory B-ALL in adults and children. As tisagenlecleucel targets CD19, there are theoretical concerns that blinatumomab pretreatment will make patients ineligible for tisagenlecleucel therapy by potentially selecting for CD19 negative populations [5]. While the global ELIANA trial excluded patients with prior blinatumomab exposure, multiple phase I and phase II trials have described the feasibility, safety and even efficacy of anti-CD19 CAR-T therapy after blinatumomab use [6–8]. In a Phase I study from the University of Pennsylvania [6] using anti-CD19 CAR-T (CD19 CAR-T) in children and young adults, 4 of 5 patients who were previously refractory to blinatumomab achieved complete remission (CR) with a *41BB* CD19 CAR-T but 3 patients subsequently relapsed with CD19 negative disease. Also, a single center study from Memorial Sloan Kettering Cancer Center (MSKCC) reported infusion of CD28z CD19 CAR-T in 13 (25%) patients previously treated with blinatumomab, and 9 of the 13 achieved CR after receipt of CAR-T [7]. Although CD19 CAR-T and blinatumomab target the same antigen, blinatumomab activity is constrained by the number and function of endogenous T cells, in contrast to CAR-T, which constitutively express a CD19-specific receptor and undergo prompt and robust in vivo expansion. It has also been reported that blinatumomab activity is partially restricted to regulatory T-cell (Treg) numbers, and those with high Tregs in peripheral blood show increased risk of failure to respond to blinatumomab. Lymphodepleting therapy with cyclophosphamide and fludarabine, may reduce Treg numbers [9]. Hence, prior use of blinatumomab (irrespective of the response) in our opinion is not considered an absolute contraindication for the use of tisagenlecleucel, however the CD19 status of the relapsed/refractory B-ALL (flow cytometry on bone marrow sample) should be considered in the decision to prescribe tisagenlecleucel, as the impact on remission durability amongst those with pre-treatment blinatumomab is not firmly established.

Inotuzumab ozogamicin (IO) is an anti-CD22-calicheamicin conjugate approved for the treatment of adults with relapsed or refractory B-ALL [10]. Since tisagenlecleucel targets CD19, prior IO use should not diminish the efficacy of anti CD19 CAR-T therapy, hence previous use of IO should not be considered an exclusion for anti CD19 CAR-T therapy. However, it is important to note that IO induces a period of B-cell aplasia following treatment by targeting CD22 on both normal and malignant B-cells. The effect that this may have on the expansion and persistence of CAR-T by reducing the number of CD19 positive cells that stimulate the growth of tisagenlecleucel is unknown. Studies of another anti CD19 CAR-T for the treatment of pediatric leukemia have shown that patients with fewer than 15% CD19 positive cells in the bone marrow at the time of infusion had shorter persistence of functional CAR-T as measured by the duration of B-cell aplasia [11].

## What is the optimal strategy to manage bridging chemotherapy and administer lymphodepleting chemotherapy between T-cell collection and infusion of CAR-T?

### Bridging chemotherapy

Given the current clinically approved indications for anti CD19 CAR-T, patients referred for CAR-T therapy are often in active relapse or have refractory disease. Therefore, chemotherapy after T-cell collection by leukapheresis is usually required to control disease until the manufacturing of CAR-T is complete. Bridging chemotherapy should focus on disease control rather than remission induction while minimizing organ toxicity or risk of infections. Of 92 patients enrolled on the ELIANA trial, 10 patients had significant adverse events or death between T-cell harvest and CAR-T infusion preventing the receipt of tisagenlecleucel. Among 75 patients who received a CAR-T infusion, 65 (87%) were treated with bridging chemotherapy between enrollment and infusion [2]. Table 1 describes various chemotherapy considerations for bridging therapy and how to manage them in relation to CAR-T infusion. The choice of bridging therapy depends on a patient’s previous treatment history and should be timed and coordinated closely with the CAR-T manufacturing / treating institution to avoid delays in the infusion of CAR-T therapy. As mentioned above, previous use of blinatumomab is not an absolute contraindication for receiving anti CD19 CAR-T therapy, however to minimize the risk of antigen loss and until more robust data are available, this therapy should ideally be avoided as a bridging chemotherapy.

**Table 1:** Suggested treatment options for bridging chemotherapy or radiotherapy between leukapheresis and CAR-T therapy

Bridging chemotherapy considerations	Time line to stop chemotherapy/radiotherapy
Systemic steroids, hydrea, and tyrosine kinase inhibitors	STOP > 3 days prior to to tisagenlecleucel infusion
Systemic chemotherapy	STOP > 1 week prior to tisagenlecleucel infusion (No drug should be administered concomitantly or following lymphodepleting chemotherapy)
►vincristine	
►6mercaptopurine	
►6-thioguanine	
►methotrexate <25 mg/m^2^	
►cytosine arabinoside <100 mg/m^2^/day	
►asparaginase (nonpegylated)	
CNS disease prophylaxis	
Salvage chemotherapy e.g.,	STOP > 2 weeks prior tisagenlecleucel infusion
►cytosine arabinoside >100 mg/m^2^, anthracyclines,	
►cyclophosphamide, methotrexate ≥25 mg/m^2^	
Radiation therapy at non-CNS site	
Pegylated asparginase	STOP > 4 weeks prior to tisagenlecleucel infusion
Anti-T Cell antibodies CNS directed radiation	STOP > 8 weeks prior to tisagenlecleucel infusion

Avoid therapies (in particular–clofarabine) which are likely to significantly impart lymphocyte number and/or function

### Lymphodepleting chemotherapy prior to infusion of CAR-T

Preclinical studies have shown that lymphodepleting chemotherapy immediately before CAR-T infusion reduces tumor burden while enhancing CAR-T expansion and persistence, likely by depletion of conventional and Tregs, and increasing cytokines driving homeostatic expansion. If there is a potential for an anti-CAR-T immune response, this may be reduced as well [12]. The ELIANA trial used a moderate dose regimen of fludarabine (30 mg/m^2^ intravenously daily for 4 days) and cyclophosphamide (500 mg/m^2^ intravenously daily for 2 days starting with the first dose of fludarabine) followed by infusion of CAR-T, 2 to 14 days after completion of lymphodepleting chemotherapy. While not studied in a randomized fashion, clinical data has generally supported the importance of lymphodepleting chemotherapy as well. In the ELIANA trial, three patients did not receive lymphodepleting chemotherapy due to leukopenia but their outcomes were not separately reported. In another clinical trial of 30 patients with relapsed/refractory B-ALL conducted at the Fred Hutchinson Cancer Research Center (FHCRC), 17 patients who received fludarabine and cyclophosphamide based lymphodepletion had improved persistence of CAR-T and better disease free survival compared to 13 patients who received either cyclophosphamide alone or cyclophosphamide with etoposide based lymphodepletion [13]. Similarly, a clinical trial of 53 patients conduced at the NCI for pediatric and young adults with relapsed/refractory ALL used different lymphodepleting chemotherapy regimen over the course of the clinical trial and based it on the disease burden at time of enrollment. There was no benefit to adjusting the lymphodepleting chemotherapy regimen based on disease burden, but overall survival in subjects receiving a fludarabine/cyclophosphamide regimen was significantly longer compared to those who received an alternative regimen [14].

Conversely, a handful of patients have received CAR-T therapy on clinical trials without antecedent lymphodepletion, including the first pediatric ALL patient successfully treated with CD19 CAR-T [15]. In the NCI adult CAR-T trial, 4 of 5 patients with B-ALL treated with donor-derived CAR-T therapy without antecedent lymphodepletion achieved measurable residual disease negative (MRD-negative) CR [15].

Currently, we recommend that patients receive fludarabine/cyclophosphamide lymphodepleting chemotherapy prior to tisagenlecleucel infusion as detailed in the package insert of the approved product and adherence to timing between lymphodepleting chemotherapy and CAR-T infusion is important. It may be appropriate for some patients with low lymphocyte counts and/or pancytopenia from disease or prior therapy to forgo lymphodepleting chemotherapy on a risk-benefit basis, with knowledge that outcomes data for this subgroup of patients is limited. Myeloid growth factors, particularly granulocyte-macrophage colony-stimulating factor (GM-CSF), are not recommended during the first 3 weeks after KYMRIAH infusion or until CRS has resolved.

## Are CAR-T a bridging therapy to allo-HCT or sufficient alone as definitive relapse therapy?

For a patient who has never undergone allo-HCT and enters a CR after anti-CD19 CAR-T, a critical question is whether to consolidate the CR with allo-HCT. CAR-T therapy, while effective, and durable in subgroup of patients, does not prevent antigen negative escape.

The NCI conducted a Phase I trial for children and young adults with relapsed/refractory CD19 positive or CD22 positive ALL to be treated with anti CD19 or anti-CD-22 CAR-T protocols, respectively. Fifty two patients were enrolled on anti CD19 and 33 patients on anti-CD22 CAR-T trial [16]. The anti-CD19 CAR-T construct utilized a CD28 costimulatory domain; while the anti-CD22 CAR-T incorporated *41BB*. In the most recent updates presented at 2018 BMT Tandem Meetings, 51 patients attained a CR, of whom 43 were MRD-negative by flow cytometry and 25 subsequently underwent allo-HCT. In a competing risks analysis of relapse versus transplant related mortality, and limited to first allo-HCT after CAR-T, it was shown that the 24-month cumulative incidence of post allo-HCT relapse was 13.5% (95% CI: 3.2–32.1%) after anti CD19 CAR-T and 11.3% (95% CI: 1.7–31.1%) after *CD22* CAR-T. These two non-randomized trials suggest the potential synergistic role of CAR-T therapy with allo-HCT to improve leukemia free survival, prior to emergence of antigen negative leukemia, without an increased risk of severe graft-versus-host disease (GVHD) [16].

In contrast, among 17 of 53 adults who underwent allo-HCT at MSKCC after anti CD19 CAR-T with a *CD28* co-stimulatory domain, five were alive in CR, six relapsed, and six died from transplant-related mortality. On further evaluation of the 32 patients with MRD-negative CR, there was no significant difference in overall survival (OS) between patients who underwent allo-HCT and those who did not (*P* = 0.89) [7].

To further elucidate the importance of disease burden pre-CAR-T therapy, patients were divided in low disease burden (<5% bone marrow blasts) and high disease burden (≥5% bone marrow blasts or extra-medullary disease). Patients with low disease burden at time of T-cell infusion had significantly longer event-free survival and OS than those with a high disease burden. The median OS among patients with a low disease burden was 20.1 months (95% CI, 8.7 to not reached), as compared with 12.4 months (95% CI, 5.9 to 20.7) among those with a high disease burden (*P* = 0.02). The significant difference in OS according to disease burden remained unaffected even when allo-HSCT after CAR-T therapy was included in the analysis. Because of the non-randomized nature of the trial, it is impossible to determine whether patients who were considered at greater risk of relapse were among those treated with allo-HCT as consolidation.

In the ELIANA trial which infused 75 patients using the anti CD19 CAR-T (with a *41BB* costimulatory domain), only eight patients (9%) underwent allo-HCT while in remission, including two patients with MRD-positive bone marrow, and two with B-cell recovery within 6 months after infusion. All eight patients who underwent allo-HCT were alive at last follow up, 4 with no relapse, and 4 with no unknown disease status. This cohort, therefore, is largely representative of outcomes after tisagenlecleucel without subsequent therapy, which notably includes only a population that is blinatumomab naïve. In the most recent update presented at American Society of Hematology 2018, the relapse-free rate was 80% at 6 months and 66% at 12 and 18 months [17]. Overall survival was nearly identical whether patients were censored at the time of SCT or not. The durability of the clinical response was associated with persistence of tisagenlecleucel in peripheral blood and persistent B-cell aplasia which is the biological correlate of ongoing CAR-T cell activity.

In the absence of long-term follow-up and given the small number of patients in the above studies with many differences including the CAR-T construct, co-stimulatory domain, conditioning therapy and previous allo-HCT, we must consider that this is an open question. In patients who have not had a prior allo-HCT, this follow on therapy must at least be considered. On the other hand, in patients who have relapsed after prior allo-HCT, the utility of second allo-HCT is also quite unclear. At this point, with studies still underway to determine who might benefit from post-CAR-T consolidation with an allo-HCT, we suggest evaluating individual patient factors (quality of available donor, comorbidities), disease related factors (MRD status, and B cell aplasia), and CAR-T related factors (co-stimulatory domain and potential persistence of CAR-T) when considering allo-HCT following anti CD19 CAR-T therapy for a patient who is allo-HCT naïve.

## What are the key considerations in using CAR-T after allo-HCT?

Prior to the availability of CAR-T therapy, there is no standard approach for treatment of a patient with relapsed/refractory B-ALL after allo-HCT and outcomes remain dismal in this setting. Donor lymphocyte infusion (DLI) and withdrawal of immune suppression result in <10% CR rates and most remissions are transient [18]. Adoptive transfer of autologous anti CD19 CAR-T has demonstrated the potency of this therapy in patients with relapsed/refractory B-ALL with a potential for long-term disease control. 61% of patients enrolled to ELIANA study had relapsed/refractory disease after allo-HCT. Generally, T-cells were successfully collected from post-HCT recipients and most were most likely of donor origin. High remission rates were seen in these studies and GVHD was not a significant treatment-related toxicity [2, 7, 8]. To clarify terminology, even though these T-cells were derived from an allogeneic graft, the cells are “autologous” because they are collected from the patient who will receive the CAR-T therapy.

There has been only one case each of acute and chronic GVHD reported in autologous CAR-T cell trials, both in the setting of trials using defined compositions of T cells. In a study from FHCRC [13], CAR-T products defined by a 1:1 ratio of CD4:CD8 cells were administered to 27 adult patients, 11 of which were engineered from engrafted fully chimeric recipients. Acute GVHD did not arise after CAR-T infusion; one patient with stage 1 acute skin GVHD before study enrollment developed chronic GVHD requiring corticosteroid therapy 3 months after CAR-T infusion. Similarly, the group from Seattle Children’s Research Institute [11] used a defined ratio of 1:1 CD4: CD8 CD19 CAR-T cells in patients with B-ALL. Among 27 of 45 patients who had previously undergone allo-HCT, only one developed grade 3 acute skin GVHD following CAR-T infusion. This patient had a prior history of GVHD and discontinued immunosuppression 1 year before CAR-T treatment.

There have been four published studies [15, 19–21] of donor-derived anti CD19 CAR-T therapy with three studies using a CD28 costimulatory domain and one with a *41BB* co-stimulatory domain including one study where donor-derived multi-virus specific T-cells were transduced with a *CD19-CAR28z* construct to prevent and treat viral infection and relapse after allo-HCT [21]. Smith et al. compiled the data for 49 patients from these four trials and reported that in patients who received donor-derived DLI, the total incidence of GVHD was 14% (*n* = 7), with an incidence of acute and chronic GVHD of 8% (*n* = 4) and 6% (*n* = 3), respectively [22]. The GVHD incidence appeared to be lower when recipient-derived (though largely likely of donor origin) rather than donor-derived CAR-T were used, suggesting that recipient-derived donor T cells were likely tolerized. It is important to note that tisagenlecleucel is an autologous product and the option to manufacture from the donor is not available. Taken together, the findings suggest that previous allo-HCT does not impact outcomes after recipient derived anti CD19 CAR-T therapy, making CAR-T therapy an excellent option for relapse after allo-HCT.

For patients who have relapsed after allo-HCT and are evaluated for CAR-T therapy, it is suggested to stop any systemic drug used to prevent or treat grade 2–4 acute GVHD or extensive chronic GVHD at least 2 weeks prior to leukapheresis (Fig. 2). The ELIANA trial allowed topical steroids for localized treatment of GVHD. It is important to note that if grade 2 or higher acute GVHD or severe chronic GVHD develop after leukapheresis, the patient should not undergo CAR-T infusion, and the leukapheresis product or resulted manufactured CAR T-cells should not be used. Systemic therapy for GVHD after CAR-T infusion, being T cell directed, would terminate the therapeutic effect.

## How is cytokine release syndrome diagnosed and what are the treatment considerations?

Key CRS manifestations include fever (92%), hypotension (67%), hypoxia (20%), and tachycardia (30%), but may also be associated with hepatic, renal, and cardiac dysfunction, and coagulopathy [2]. CRS begins with fever, and onset of severe CRS is most often indicated by unstable hypotension. Cytokine release syndrome (CRS), including fatal or life-threatening reactions, occurred following treatment with tisagenlecleucel in 54 (79%) of 68 pediatric and young adult patients with relapsed/refractory B-ALL. CRS severity was at least grade 3 (Penn grading system) in approximately half (49%) of all patients with a median time to onset of 3 days (range: 1–51 days); only in two patients did CRS begin after day 10. CRS resolution occurred at a median of 8 days (range: 1–36). Twenty-seven (50%) of the 54 patients with CRS were treated with tocilizumab; 7 (13%) received two doses, 3 (6%) received three doses, and 14 (26%) also received additional corticosteroids. Two deaths occurred within 30 days of tisagenlecleucel infusion, one due to CRS and progressive leukemia, and the other due to intracranial hemorrhage in the context of resolving CRS with abdominal compartment syndrome, coagulopathy, and renal failure.

Risk factors for severe CRS in this patient population are high pre-infusion tumor burden (>50% marrow blasts), leukemia growing through lymphodepleting chemotherapy, high T cell dose, active infections, and/or other inflammatory processes. As a result, patients with rapidly accelerating extramedullary disease despite lymphodepleting chemotherapy, or patients with active infection, are likely to not be candidates for this therapy.

In the ELIANA trial, the Penn grading scale was used to diagnose CRS. Currently, multiple CRS grading system have been used in various clinical trials including the NCI Common Terminology Criteria for Adverse Events (CTCAE) system, Lee et al. [23]. and Neelapu et al. [24]. scales, all of which have advantages and disadvantages. Given the need for uniformity and replicability, ASBMT has developed a harmonized CRS grading scale for toxicity assessments, thus readers are directed to that paper for assessments and exact definitions of toxicity of CAR-T cells [25].

Because of the risk of CRS and neurological toxicities, KYMRIAH (tisagenlecleucel) is available only through a restricted program under a REMS called the KYMRIAH REMS. Two doses of tocilizumab must be available on site for each patient prior to infusion of CAR-T therapy. Monitoring patients for signs or symptoms of CRS for at least 4 weeks after treatment with tisagenlecleucel is necessary. When patients are treated in the outpatient setting, patients must remain within 1–2 h of the hospital and to seek immediate medical attention should signs or symptoms of CRS occur at any time. At the first sign of CRS (fever), immediate hospitalization is imperative to allow prompt institution of treatment with supportive care, tocilizumab and/or corticosteroids (as indicated). When patients are admitted for fever, immediate management is focused in febrile neutropenia.

## How do we diagnose and manage neurotoxicity after CAR-T therapy?

Neurotoxicity is an acknowledged challenge after CAR-T, however in contra-distinction to CRS, the exact pathogenesis and optimal management of CAR-T mediated neurologic toxicities remains to be better defined and studied in animal models [26, 27]. The onset of neurological toxicity is most commonly concurrent with or following resolution of CRS although it can also occur in the absence of CRS. The most common neurological toxicities observed with tisagenlecleucel include headache (37%), encephalopathy (34%), and delirium (21%) [2]. Neurological toxicities including severe or life-threatening reactions occurred in 49 (72%) of 68 patients with relapsed/refractory B-ALL following treatment with tisagenlecleucel including at least grade 3 events in 21% of patients. Eighty-eight percent of these neurological toxicities occurred within 8 weeks following tisagenlecleucel infusion and were managed primarily with supportive care; no cerebral edema has been reported. Very few patients received steroids for neurological toxicities. Median time to the first event was 6 days from infusion (range: 1–359), and the median duration was 6 days with resolution occurring within 3 weeks for 79% of patients. The consensus neurotoxicity grading system does not include headache as part of the grading system, since headache is nearly universal in patients with CRS.

When managing neurotoxicity, it is important to exclude alternative etiologies, especially infectious causes, stroke or hemorrhage. The extent of work-up (e.g., magnetic resonance imaging, electroencephalogram, cerebrospinal fluid analysis in selected patients) can be tailored to the severity and nature of symptoms. Imaging may also be useful to detect cerebral edema, which has been reported with other anti CD19 CAR T-cell products used for the treatment of B-ALL but has not been reported with tisagenlecleucel to date [2, 8, 11, 28, 29]. Patients with a negative work-up may be treated expectantly. Levetiracetam or alternative anti-seizure prophylaxis for patients with a history of seizures or a history of prior neurotoxicity should be considered. In the ELIANA trial, 10 (13%) patients with CNS-2 disease (CSF containing blasts, but <5 WBCs/microliter) received tisagenlecleucel. Neurotoxicity did not appear to be more severe in patients with CNS involvement than in those with bone marrow involvement only. Patients with frank CSF leukemia should not receive CAR-T cells until central nervous system (CNS) disease is improving or completely resolved.

## How should we monitor response after CAR-T therapy?

Initial response assessment after CAR-T therapy for B-ALL should occur 3–4 weeks after CAR-T. Patients with B-ALL who do not respond within 4 weeks of CAR-T therapy are unlikely to respond, although resolving MRD-level disease has been seen in a small number of patients. Response assessments after CAR-T therapy are similar to response assessments performed after traditional chemotherapy for B-ALL and should include complete blood count with differential, bone marrow biopsy and aspirate, and a lumbar puncture (with or without a history of CNS disease). Assessment for MRD should be performed when available due to its prognostic importance. There are advantages and disadvantages of both Immunoglobulin next generation sequencing (NGS) and flow cytometry assays for detection of MRD. A flow cytometry assay for MRD is useful to identify surface markers (particularly CD19, but also CD22, and CD20) with therapeutic implications, while NGS based MRD may be more sensitive to detect lower levels of disease and has been strongly correlated with outcome after tisagenlecleucel [13]. When using flow cytometry assay to identify MRD, it is important to notify the laboratory performing MRD assessment that the patient received a CD19 targeted product and is at risk for a CD19-negative relapse which may impact design and interpretation of flow cytometry and possibly selection of future therapies.

CAR-T can cross the blood brain barrier and have potential to target tumor cells within the CNS and provide ongoing leukemia surveillance. For this reason, during CNS restaging after anti CD19 CAR-T, administration of prophylactic intrathecal chemotherapy, due to its potential to kill CAR-T cells in this compartment, is not recommended. Of note, it is important to recognize that morphologically abnormal cells observed in CSF may be due to CAR-T cells (and not leukemia). In those situations, flow cytometry may be useful to differentiate CAR-T cells from residual lymphoblasts when there is a concern.

Patients who achieve MRD-negative CR often have incomplete recovery of peripheral blood counts at the time of response assessment [2, 7, 8, 13, 28]. The reasons for this are multifactorial including heavily pretreated bone marrow, choice and intensity of bridging and/or lymphodepleting chemotherapy before CAR-T, and likely bone marrow suppression due to CAR-T expansion and resulting cytokine elevations. Incomplete count recovery at 4 weeks (even up to 3 months in some instances) should not be considered a negative prognostic sign. Most patients will slowly recover blood counts over subsequent weeks or months.

Patients who have an initial response to therapy are at risk for relapse with either CD19-positive (often due to loss of CD19 CAR-T persistence) or CD19-negative (emergence of a resistant clone) disease. Ongoing monitoring for relapse should be individualized. For a patient that does not receive further therapy after anti CD19 CAR-T, a reasonable monitoring approach is complete blood count with differential, and bone marrow biopsy and aspirate with assessment for MRD every 3 months for the 6–12 months after treatment. Obtaining peripheral blood B-cell counts monthly for the first 6 months after treatment may allow detection of early loss of B-cell aplasia, which has been associated with an increased risk of relapse and may be used to stratify which patients are treated with subsequent allo-HCT and/or offered enrollment on clinical trials of reinfusion of CAR T-cells. Many trials use marrow surveillance out to a year, but at the very least, a marrow at 3 months is an excellent way to screen for hematogones, an indicator of early B cell recovery. This in turn may inform a decision to reinfuse CAR-T cells or pursue allo-HCT. The frequency of CSF evaluation can be individualized.

## How do we manage B cell aplasia and hypogammaglobulinemia after CD19-targeted CAR-T cell therapy?

Specificity of CAR-T for target antigen is high; therefore, few off-target toxicities have been reported. Cytopenias following CAR-T cell therapy may be prolonged and have been associated with late infectious complications including fatal encephalitis and systemic mycosis. In the ELIANA trial, 53% of patients had grade 3 or 4 neutropenia that had not resolved by 28 days after infusion and 41% had grade 3 or 4 thrombocytopenia. 66% of these patients had resolution of neutropenia and 73% had resolution of thrombocytopenia to at least grade 2 by month 3. Use of myeloid growth factors is not recommended within the first 3 weeks after treatment or until CRS has resolved but should be considered for patients with prolonged neutropenia.

Conversely, on-target, but off-tumor toxicities are common. For anti CD19 CAR-T therapy, B-cell aplasia is nearly universal following successful treatment and can be used as a pharmacodynamic marker of functional CAR-T cell persistence [2, 8]. Resultant hypogammaglobulinemia has been reported in 43% of children treated with tisagenlecleucel, but true rates of hypogammaglobulinemia are likely higher due to empiric immunoglobulin replacement in some patients before the development of laboratory-confirmed hypogammaglobulinemia [2, 29]. Children have usually been treated with empiric immunoglobulin replacement similar to patients with X-linked agammaglobulinemia for the duration of functional CAR-T persistence, which appears to mitigate most acute infectious complications. Whether this is required in adult patients who may have a greater repertoire of antibodies produced by pre-existing CD19-negative plasma cells is not yet clear.

Prospective data on immunoglobulin replacement after CD19-targeted CAR-T cell therapy is currently unavailable, however, as severe hypogammaglobulinemia may increase risk for respiratory tract and other infections with encapsulated bacterial organisms (e.g., Streptococcus pneumoniae and Haemophilus influenzae type b), testing of serum IgG levels frequently after CD19-targeted CAR-T therapy, and administration of intravenous immunoglobulins to keep levels of IgG > 400 mg/dL may be considered until long term data on the association of hypogammaglobinemia with infections is well established. Patients with prolonged (>6 months) B cell aplasia have been managed with subcutaneous immunoglobulin products, which can be administered at home and result in higher steady state IgG levels. Since prospective data on immunoglobulin replacement dosage is unavailable currently, institutional policies should be followed if replacement is undertaken. Long term follow-up will be required to understand the long-term effects of B-cell aplasia in both children and adults.

## What are the late effects of CAR-T therapy?

Most patients participating in CAR-T trials have been followed for one or two years after treatment. Thus, the ability to assess the risk of long-term adverse events and late toxic effects of CAR-T, including tisagenlecleucel, is currently limited, although some potential late effects are theoretically predictable. To date, the main concerns regarding potential long-term sequelae of CAR-T include persistent cytopenia, prolonged B cell aplasia with acquired hypogammaglobulinemia, risks for infections, secondary malignancies, and new incidence or exacerbation of neurologic or autoimmune disorders [30, 31].

While there is a theoretical risk for potentiation of alloreactive T-cells in patients who have undergone prior allo-HCT and still have donor T-cell engraftment, to date, development of GVHD directly as a result of CAR-T has not been reported despite>50% of patients having prior allo-HCT in clinical trials of tisagenlecleucel for pediatric leukemia [2, 8, 11, 28, 29]. It is notable that these trials excluded patients with significant GVHD and required a washout period off immunosuppression prior to apheresis. Attention should be paid to these criteria when patients are considered for commercial CAR-T cell therapy.

### Infections

Patients treated with anti CD19 CAR-T for B-cell hematologic malignancies are at high risk of infection due to prior cytotoxic treatments, development of CRS, the risk for prolonged cytopenia and B-cell aplasia with associated hypogammaglobulinemia. Thus, antimicrobial prophylaxis is recommended depending on institutional guidelines for CAR-T cells. (Table 2) Potential regimens include: levofloxacin for gram-negative bacteria prophylaxis while patients are neutropenic, fluconazole or micafungin for *Candida* species prophylaxis while patients are neutropenic, trimethoprim-sulfamethoxazole (or pentamidine if allergic) for pneumocystis jiroveci prophylaxis for ≥3 months after CD19-targeted T cell therapy, and acyclovir for herpes simplex virus and varicella zoster virus prophylaxis for ≥3 months after CD19-targeted T cell therapy [32, 33].

**Table 2:** Infection prophylaxis for patients undergoing anti CD-19 CAR-T therapy

Infection	Prophylaxis and duration^a^
Gram-negative bacteria	Levofloxacin (while patients are neutropenic)
*Candida* species	Fluconazole or micafungin
Pneumocystis jiroveci	Trimethoprim-sulfamethaxazole (or pentamidine if allergic) for ≥3 months after CD19 targeted T cell therapy
Herpes simplex virus and varicella zoster virus	Acyclovir or valacyclovir for ≥3 months after CD19 targeted T cell therapy

^a^For patients with allergies to particular medications, substitute with appropriate alternatives per institutional guidelines

Approximately 20–40% of patients develop infections within the first month after CAR-T therapy despite antimicrobial prophylaxis [32, 33]. Bacterial and viral infections are the most common, although invasive fungal infections have also been described. Clinically significant reactivation of latent DNA viruses (e.g., cytomegalovirus, Epstein-Barr virus, BK polyomavirus) do not appear to be common, albeit prospective screening studies are lacking.

The incidence and severity of late infections after CAR-T therapy are not well described. Limited data suggest a low incidence of late infections, most of which are mild and due to respiratory viruses [32–34]. Careful monitoring for other infections is warranted as clinical use of CD19-directed CAR-T therapy expands to include more patients with chronic infections, such as hepatitis B, which may reactivate in this clinical setting. At this time there are data to regarding HBV reactivation after CD19-directed CAR-T therapy, and there is no data to suggest HBV reactivation prophylaxis. At this time safety data for CAR-T therapy in human immunodeficiency virus (HIV) affected patients are not yet available, as this patient population has been excluded from CAR-T clinical trials. Of notice, some screening tests for HIV may lead to false positive results in case lentiviral vectors are used to produce CART cells.

### Vaccinations

At this time there are no data to guide vaccination in this patient population. Studies of patients with B-cell depletion after rituximab have demonstrated the potential ability to mount responses to vaccines, particularly >6 months after therapy and when conjugated vaccines are used, even in the absence of measurable peripheral blood B-cells [35]. Limited evidence from patients after anti CD19 targeted CAR-T therapy suggest that the preexisting levels of antibodies may not be affected given that antigen-specific IgG may be produced by long-lived plasma cells that do not express surface CD19 [36]. However, the B cell aplasia in CAR-T patients can be absolute, and there is no IgA or IgM (especially in children), questioning the efficacy of vaccinations in these populations, thus might need to wait until there is evidence of B cell recovery before commencing vaccinations.

While there is no data to guide vaccination in this patient population is available, guidelines from the Infectious Disease Society of America for immunocompromised or cancer patients and the US Advisory Committee on Immunization Practices may be considered in individuals with a complete response for ≥6 month [37, 38]. However, many groups are waiting for resolution of B-cell aplasia before restarting vaccination. IVIg products supply antibody protection in patients with B-cell aplasia. Thus decision to start vaccinations while receiving IVIg should be prioritized based on institutional guidelines or on individual basis. Once vaccination decision has been made, priority should be given to inactivated influenza vaccines, the 13-valent Pneumococcal conjugate vaccine (Prevnar 13), and Haemophilus influenzae type b (Hib) conjugate vaccine. Serologic testing should ideally be obtained to evaluate the need for vaccination and to test for responses. Vaccination schedules similar to those given after HCT may be needed to elicit responses [39]. Data from ongoing observational studies would be essential in order to generate future recommendations for vaccinations after CD19-targeted CAR-T cell therapy.

### Subsequent malignancies

Retroviral and lentiviral gene transfer systems are the most commonly used vectors in the genetic modification of T-cell therapies. Vectors derived from these families of viruses come with two potential risks: (i) the production of replication-competent viruses (RCV) and (ii) insertional mutagenesis, specifically oncogenic activation. Results of RCV testing from CAR-T trials demonstrate no risk to date [40]. Similarly, a number of clinical trials with gamma-retroviral and lentiviral modified T-cells have not yielded evidence for insertional mutagenesis in T cells despite long-term persistence of transduced cells. The safety profile in studies to date show no evidence of vector-induced immortalization, clonal expansion, or enrichment for integration sites near genes implicated in growth control or transformation [41–43]. While the risk seems very low, clinical monitoring for secondary malignancies and long-term follow-up after CAR-T therapy should continue to be part of clinical trial protocols and is being requested as part of the long-term follow-up by FDA and European Medicines Agency (EMA). A post-marketing, prospective, multi-center, observational study to assess the long-term safety of tisagenlecleucel and the risk of secondary malignancies occurring after treatment with tisagenlecleucel, is required by the FDA. Per the FDA requirements the study should include at least 1000 pediatric and young adult patients with relapsed / refractory B-ALL, and the enrolled patients should be followed for 15 years [44].

### Neurologic disorders

Acute neurotoxicity after CAR-T therapy is well documented, with symptoms including confusion, delirium, expressive aphasia, obtundation, myoclonus, seizure, and cerebral edema [24, 45] as described earlier in this manuscript. The exact pathophysiology of CAR-T induced neurotoxicity is unclear, but two explanations have been suggested (i) passive diffusion of cytokines, such as IL-6 and IL-15, through the blood brain barrier, and (ii) trafficking of the CAR-T into the CNS [24]. Disruption of the blood–brain barrier may also be a contributory factor [45]. Although most cases of neurological toxicity seem reversible, data regarding long term neurological sequelae of CAR-T therapy are not available. Until data is available, patients who developed acute neurotoxicity after CAR-T infusions should be vigilantly monitored with history and complete neurologic exam. Furthermore, there should be a low threshold for performing neurocognitive testing if cognitive impairment is detected, noting that these patients will have received other chemotherapy and/or HCT as well. No data are currently available regarding late autoimmune disorders.

## Conclusions

Data regarding CAR-T late effects are still limited. Detection of these effects requires ongoing long-term follow-up and enhanced clinical awareness by clinicians caring for patients after CAR-T therapy worldwide. A 15-year follow-up is requested as part of the marketing authorization of tisagelecleucel and axicabtagene ciloleucel, both by FDA and EMA. Continental registries such as CIBMTR or EBMT may become essential tools for this endeavor, helping to capture infrequent and delayed events, including the outcome of pregnancies in patients or partners. We hope that this paper will be helpful for the clinical management of issues revolving around CAR-T and allo-HCT in relapsed/refractory B-ALL patients.

## References

[1] June CH, Sadelain M (2018). Chimeric antigen receptor therapy. N Engl J Med.

[2] Maude SL, Laetsch TW, Buechner J, Rives S, Boyer M, Bittencourt H (2018). Tisagenlecleucel in children and young adults with B-Cell lymphoblastic leukemia. N Engl J Med.

[3] Maus MV, Nikiforow S (2017). The why, what, and how of the new FACT standards for immune effector cells. J Immunother Cancer.

[4] Das Rajat SJ, Barrett D. T cell dysfunction in pediatric cancer patients at diagnosis and after chemotherapy can limit chimeric antigen receptor potential. Cancer Res. 2018;2018(78 (13 suppl)):Abstract nr 1631.

[5] Mejstrikova E, Hrusak O, Borowitz MJ, Whitlock JA, Brethon B, Trippett TM (2017). CD19-negative relapse of pediatric B-cell precursor acute lymphoblastic leukemia following blinatumomab treatment. Blood Cancer J.

[6] Grupp S, Maude S, Shaw P, Aplenc R, Barrett D, Callahan C, et al. Durable Remission in Children with relapsed/refractory ALL treated with T cells engineered with a CD19 - Targeted Chimeric Antigen Receptor (CTL019). In: American Society of Hematology: Blood, 2015. p 681.

[7] Park JH, Riviere I, Gonen M, Wang X, Senechal B, Curran KJ (2018). Long-term follow-up of CD19 CAR therapy in acute lymphoblastic leukemia. N Engl J Med.

[8] Lee DW, Kochenderfer JN, Stetler-Stevenson M, Cui YK, Delbrook C, Feldman SA (2015). T cells expressing CD19 chimeric antigen receptors for acute lymphoblastic leukaemia in children and young adults: a phase 1 dose-escalation trial. Lancet.

[9] Duell J, Dittrich M, Bedke T, Mueller T, Eisele F, Rosenwald A (2017). Frequency of regulatory T cells determines the outcome of the T-cell-engaging antibody blinatumomab in patients with B-precursor ALL. Leukemia.

[10] Kantarjian HM, DeAngelo DJ, Stelljes M, Martinelli G, Liedtke M, Stock W (2016). Inotuzumab Ozogamicin versus standard therapy for acute lymphoblastic leukemia. N Engl J Med.

[11] Gardner RA, Finney O, Annesley C, Brakke H, Summers C, Leger K (2017). Intent-to-treat leukemia remission by CD19 CAR T cells of defined formulation and dose in children and young adults. Blood.

[12] Davila ML, Kloss CC, Gunset G, Sadelain M (2013). CD19 CAR-targeted T cells induce long-term remission and B Cell Aplasia in an immunocompetent mouse model of B cell acute lymphoblastic leukemia. PLoS One.

[13] Turtle CJ, Hanafi LA, Berger C, Gooley TA, Cherian S, Hudecek M (2016). CD19 CAR-T cells of defined CD4+:CD8+composition in adult B cell ALL patients. J Clin Invest.

[14] Lee DW, Stetler-Stevenson M, Yuan C, Shah N, Delbrook C, Yates B, et al. Long-Term Outcomes Following CD19 CAR T Cell Therapy for B-ALL Are Superior in Patients Receiving a Fludarabine/Cyclophoshamide Preparative Regimen and Post-CAR Hematopoietic Stem Cell Transplantation. In: American Society of Hematology: Blood, 2016. p 218.

[15] Brudno JN, Somerville RP, Shi V, Rose JJ, Halverson DC, Fowler DH (2016). Allogeneic T cells that express an Anti-CD19 chimeric antigen receptor induce remissions of B-Cell malignancies that progress after allogeneic hematopoietic stem-cell transplantation without causing graft-versus-host disease. J Clin Oncol.

[16] Shalabi H, Delbrook C, Stetler-Stevenson M, Yuan C, Steinberg S, Yates B, et al. Chimeric Antigen Receptor T-Cell Therapy Can Render Patients with ALL into PCR-Negative Remission and Can be an Effective Bridge to Transplant. In: BMT Tandem Scientific Meeting. Salt Lake City, Utah, 2018.

[17] Grupp Stephan A., Maude Shannon L., Rives Susana, Baruchel Andre, Boyer Michael W., Bittencourt Henrique, Bader Peter, Büchner Jochen, Laetsch Theodore W., Stefanski Heather, Myers Gary Douglas, Qayed Muna, Pulsipher Michael A., De Moerloose Barbara, Yanik Gregory A., Davis Kara L., Martin Paul L., Nemecek Eneida R., Peters Christina, Krueger Joerg, Balduzzi Adriana, Boissel Nicolas, Mechinaud Francoise Marie, Leung Mimi, Eldjerou Lamis K., Yi Lan, Mueller Karen Thudium, Bleickardt Eric, Hiramatsu Hidefumi (2018). Updated Analysis of the Efficacy and Safety of Tisagenlecleucel in Pediatric and Young Adult Patients with Relapsed/Refractory (r/r) Acute Lymphoblastic Leukemia. Blood.

[18] Fielding AK, Richards SM, Chopra R, Lazarus HM, Litzow MR, Buck G (2007). Outcome of 609 adults after relapse of acute lymphoblastic leukemia (ALL); an MRC UKALL12/ECOG 2993 study. Blood.

[19] Dai H, Zhang W, Li X, Han Q, Guo Y, Zhang Y (2015). Tolerance and efficacy of autologous or donor-derived T cells expressing CD19 chimeric antigen receptors in adult B-ALL with extramedullary leukemia. Oncoimmunology.

[20] Kebriaei P, Singh H, Huls MH, Figliola MJ, Bassett R, Olivares S (2016). Phase I trials using sleeping beauty to generate CD19-specific CAR T cells. J Clin Invest.

[21] Cruz CR, Micklethwaite KP, Savoldo B, Ramos CA, Lam S, Ku S (2013). Infusion of donor-derived CD19-redirected virus-specific T cells for B-cell malignancies relapsed after allogeneic stem cell transplant: a phase 1 study. Blood.

[22] Smith M, Zakrzewski J, James S, Sadelain M (2018). Posttransplant chimeric antigen receptor therapy. Blood.

[23] Lee DW, Gardner R, Porter DL, Louis CU, Ahmed N, Jensen M (2014). Current concepts in the diagnosis and management of cytokine release syndrome. Blood.

[24] Neelapu SS, Tummala S, Kebriaei P, Wierda W, Gutierrez C, Locke FL (2018). Chimeric antigen receptor T-cell therapy - assessment and management of toxicities. Nat Rev Clin Oncol.

[25] Lee Daniel W., Santomasso Bianca D., Locke Frederick L., Ghobadi Armin, Turtle Cameron J., Brudno Jennifer N., Maus Marcela V., Park Jae H., Mead Elena, Pavletic Steven, Go William Y., Eldjerou Lamis, Gardner Rebecca A., Frey Noelle, Curran Kevin J., Peggs Karl, Pasquini Marcelo, DiPersio John F., van den Brink Marcel R.M., Komanduri Krishna V., Grupp Stephan A., Neelapu Sattva S. (2019). ASTCT Consensus Grading for Cytokine Release Syndrome and Neurologic Toxicity Associated with Immune Effector Cells. Biology of Blood and Marrow Transplantation.

[26] Norelli M, Camisa B, Barbiera G, Falcone L, Purevdorj A, Genua M (2018). Monocyte-derived IL-1 and IL-6 are differentially required for cytokine-release syndrome and neurotoxicity due to CAR T cells. Nat Med.

[27] Giavridis T, van der Stegen SJC, Eyquem J, Hamieh M, Piersigilli A, Sadelain M (2018). CAR T cell-induced cytokine release syndrome is mediated by macrophages and abated by IL-1 blockade. Nat Med.

[28] Maude SL, Frey N, Shaw PA, Aplenc R, Barrett DM, Bunin NJ (2014). Chimeric antigen receptor T cells for sustained remissions in leukemia. N Engl J Med.

[29] Neelapu SS, Locke FL, Bartlett NL, Lekakis LJ, Miklos DB, Jacobson CA (2017). Axicabtagene ciloleucel CAR T-Cell Therapy in Refractory Large B-Cell Lymphoma. N Engl J Med.

[30] Zheng Ping-Pin, Kros Johan M., Li Jin (2018). Approved CAR T cell therapies: ice bucket challenges on glaring safety risks and long-term impacts. Drug Discovery Today.

[31] Bonifant CL, Jackson HJ, Brentjens RJ, Curran KJ (2016). Toxicity and management in CAR T-cell therapy. Mol Ther Oncolytics.

[32] Park Jae H, Romero F Andres, Taur Ying, Sadelain Michel, Brentjens Renier J, Hohl Tobias M, Seo Susan K (2018). Cytokine Release Syndrome Grade as a Predictive Marker for Infections in Patients With Relapsed or Refractory B-Cell Acute Lymphoblastic Leukemia Treated With Chimeric Antigen Receptor T Cells. Clinical Infectious Diseases.

[33] Hill JA, Li D, Hay KA, Green ML, Cherian S, Chen X (2018). Infectious complications of CD19-targeted chimeric antigen receptor-modified T-cell immunotherapy. Blood.

[34] Kochenderfer JN, Somerville RPT, Lu T, Yang JC, Sherry RM, Feldman SA (2017). Long-duration complete remissions of diffuse large B cell lymphoma after anti-CD19 chimeric antigen receptor T cell therapy. Mol Ther.

[35] Bingham CO, Looney RJ, Deodhar A, Halsey N, Greenwald M, Codding C (2010). Immunization responses in rheumatoid arthritis patients treated with rituximab: results from a controlled clinical trial. Arthritis Rheum.

[36] Bhoj VG, Arhontoulis D, Wertheim G, Capobianchi J, Callahan CA, Ellebrecht CT (2016). Persistence of long-lived plasma cells and humoral immunity in individuals responding to CD19-directed CAR T-cell therapy. Blood.

[37] Rubin Lorry G., Levin Myron J., Ljungman Per, Davies E. Graham, Avery Robin, Tomblyn Marcie, Bousvaros Athos, Dhanireddy Shireesha, Sung Lillian, Keyserling Harry, Kang Insoo (2014). Executive Summary: 2013 IDSA Clinical Practice Guideline for Vaccination of the Immunocompromised Host. Clinical Infectious Diseases.

[38] Kroger A, Duchin J, Vázquez M. General Best Practice Guidelines for Immunization. Best Practices Guidance of the Advisory Committee on Immunization Practices (ACIP). In. https://www.cdc.gov/vaccines/hcp/acip-recs/general-recs/index.html Accessed 12 Nov 2018.

[39] Carpenter PA, Englund JA (2016). How I vaccinate blood and marrow transplant recipients. Blood.

[40] Cornetta K, Duffy L, Turtle CJ, Jensen M, Forman S, Binder-Scholl G (2018). Absence of replication-competent lentivirus in the clinic: analysis of infused T cell products. Mol Ther.

[41] Persons DA, Baum C (2011). Solving the problem of gamma-retroviral vectors containing long terminal repeats. Mol Ther.

[42] Scholler J, Brady TL, Binder-Scholl G, Hwang WT, Plesa G, Hege KM (2012). Decade-long safety and function of retroviral-modified chimeric antigen receptor T cells. Sci Transl Med.

[43] Milone MC, O’Doherty U (2018). Clinical use of lentiviral vectors. Leukemia.

[44] Bryan W. FDA Letter - Biologics License Application. In. https://www.fda.gov/downloads/BiologicsBloodVaccines/CellularGeneTherapyProducts/ApprovedProducts/UCM574106.pdf. Accessed: 12 Nov 2018.

[45] Gust J, Hay KA, Hanafi LA, Li D, Myerson D, Gonzalez-Cuyar LF (2017). Endothelial activation and blood-brain barrier disruption in neurotoxicity after adoptive immunotherapy with CD19 CAR-T cells. Cancer Discov.

